# A chemical approach facilitates CRISPRa-only human iPSC generation and minimizes the number of targeted loci required

**DOI:** 10.2144/fsoa-2023-0257

**Published:** 2024-05-15

**Authors:** Ramzey Abujarour, Jason Dinella, Mochtar Pribadi, Lauren K Fong, Matthew Denholtz, Alma Gutierrez, Matt Haynes, Enaaya Mahmood, Tom T Lee, Sheng Ding, Bahram Valamehr

**Affiliations:** 1 Fate Therapeutics, San Diego, CA 92121, USA; 2 School of Pharmaceutical Sciences, Tsinghua University, Beijing, China

**Keywords:** CRISPR, CRISPRa, human, induced pluripotent, reprogramming, small molecules, stem cells

## Abstract

**Aim:** We explored the generation of human induced pluripotent stem cells (iPSCs) solely through the transcriptional activation of endogenous genes by CRISPR activation (CRISPRa). **Methods:** Minimal number of human-specific guide RNAs targeting a limited set of loci were used with a unique cocktail of small molecules (CRISPRa-SM). **Results:** iPSC clones were efficiently generated by CRISPRa-SM, expressed general and naive iPSC markers and clustered with high-quality iPSCs generated using conventional reprogramming methods. iPSCs showed genomic stability and robust pluripotent potential as assessed by *in vitro* and *in vivo*. **Conclusion:** CRISPRa-SM-generated human iPSCs by direct and multiplexed loci activation facilitating a unique and potentially safer cellular reprogramming process to aid potential applications in cellular therapy and regenerative medicine.

## Tweetable abstract

Combined chemical and CRISPRa-mediated approach leads to efficient generation of human iPSCs.

## Abstract (plain language summary)

Human iPSCs are conventionally derived through transient expression of exogenous reprogramming factors.

We previously generated iPSCs from murine fibroblasts using SunTag-based CRISPRa method.

With a minimal number of human-specific guide RNAs targeting a limited set of loci, combining CRISPRa with a unique cocktail of small molecules (CRISPRa-SM), bona fide iPSC colonies were successfully derived from human fibroblasts.

iPSC clones expressed general and naive iPSC markers and clustered with high-quality iPSCs generated using conventional reprogramming methods.

The CRISPRa-SM iPSCs displayed genomic stability and robust pluripotent potential as assessed by *in vitro* tri-lineage differentiation and *in vivo* teratoma assays.

The CRISPRa-SM system generated human iPSCs by direct and multiplexed loci activation facilitating a unique and potentially safer cellular reprogramming process to aid potential applications in cellular therapy and regenerative medicine.

Human-induced pluripotent stem cells (iPSCs) are equivalent to embryonic stem cells in term of their capacity to undergo tri-lineage differentiation and unlimited self-renewal, thus holding great promise in regenerative medicine and basic research [1,2]. iPSCs are typically generated by transient and forced expression of a set of exogenous reprogramming factors which are eventually lost or silenced, allowing for pluripotency to be maintained solely by the endogenous pluripotency gene circuitry [3,4]. Canonical reprogramming factors used for the generation of iPSCs include OCT4 in variable combinations with SOX2, KLF4, MYC and LIN28, which are used often with P53 inhibitors [^4-7^]. Furthermore, the use of miRNAs can enhance reprogramming into iPSCs [^8-10^].

Direct transcriptional activation of endogenous genes has been achieved using clustered regularly interspaced short palindromic repeats (CRISPR) guide RNAs (gRNAs) and deactivated Cas9 nucleases (dCas9) fused to transcription activation domains (e.g. VP64) [11,12]. Several CRISPR activation (CRISPRa) systems demonstrated variable activation capacities that were impacted by the targeted loci, cell type and species [11]. Initial attempts to use dCas9-VP64 resulted in low to moderate transcriptional activation and required the use of multiple gRNAs per locus [12]. CRISPRa was enhanced through the use of chimeric transcription activation domains [13], synergistic activation mediator (SAM) [14] and the SunTag system [15]. The SunTag system, which relies on dCas9 fused with a tandem array of SunTag peptides that recruit up to ten VP64 proteins, has been shown to be effective in transcriptional activation [15,16].

Compared with the over-expression of exogenous reprogramming factors, direct and specific transcriptional activation of the promoters and enhancers of endogenous pluripotency genes using CRISPRa may lead to a reprogramming process of higher fidelity and iPSCs of higher quality [17]. Early attempts to generate iPSCs using CRISPRa were unsuccessful. Activating endogenous reprogramming genes *OCT4*, *SOX2*, *KLF4*, *MYC* and *LIN28* using as many as 13 gRNAs and either dCas9-VP64 or the SAM system failed to reprogram human fibroblasts into iPSCs, likely due to insufficient activation of some of the targeted genes [18]. Using dCas9-VP192 (containing three VP64 copies) to activate *OCT4*, *SOX2* and *LIN28* also failed to generate iPSCs from human fibroblasts, owing to the complexity associated with the process [19]. However, the use of the same dCas9-VP192 CRISPRa system to target five reprograming genes and an Alu-motif enriched in promoters of genes expressed during embryo genome activation (EEA motif) when combined with P53 knockdown by shRNA led to a successful generation of human iPSCs [20]. An improved CRISPRa method for the generation of human iPSCs by the same group was later described and relied on the use of 15 gRNAs targeting six loci as well as miRNAs targeting the miR-302/367 cluster [17].

In this study, we report the generation of iPSCs from human fibroblasts solely using the CRISPRa SunTag system combined with a unique combination of small molecule inhibitors to facilitate and maintain the reprogramming process, CRISPRa-SM. Unlike previous strategies, CRISPRa-SM did not require exogenous reprogramming gene expression nor shRNAs/miRNAs. This was made possible by the addition of small molecule inhibitors of signaling pathways involved in the enhancement of cellular reprogramming, the maintenance of naive pluripotency and survival of iPSCs [21,22]. Using our previously described naive iPSC generation platform [^21-23^], clonal CRISPRa-SM iPSC lines were derived and showed to express canonical and naive iPSC markers and clustered with iPSCs generated using conventional reprogramming methods. The CRISPRa-SM iPSC lines maintained genomic stability and high pluripotent potential as determined by the stringent teratoma assay. Our findings highlight the potential of CRISPRa-SM to facilitate the generation of iPSCs in a manner that does not require forced expression of various transgenes.

## Materials & methods

### Cell culture

Human neonatal fibroblasts were cultured in Dulbecco's Modified Eagle's Medium (DMEM) with 10% fetal bovine serum (FBS), 1% GlutaGro, 1% non-essential amino acids (NEAA), and 1% penicillin/streptomycin. iPSCs were cultured in iPSC medium as described previously [21,22] containing small molecules Thiazovivin (5 μM, Ryss Labs), CHIR99021 (1 μM, Abcam), and PD0325901 (5 μM, Abcam). Fibroblasts and iPSC cultures were dissociated using Accutase. Fibroblasts were maintained in incubators set at 5% CO2 at 37 °C and iPSCs were maintained under the same conditions and hypoxic O2 levels (<10%).

### Plasmid design & construction

The components of the CRISPRa system have been described previously [16]. To create the lentiviral vector expressing the human gRNAs, a vector was constructed using synthesized gBlocks containing the human U6 promoter. The specific sgRNA, and appropriate homologous sequences for NEBuilder assembly into pSLQ1373-sgRNA-BFP were digested with XbaI and BamHI. Each gBlock contains a 5′ assembly linker (CAGTTTGGTTAGTACCGGGCCCGCTCTAGA), the hU6 promoter (GAGGGCCTATTTCCCATGATTCCTTCATATTTGCATATACGATACAAGGCTGTTAGAGAGATAATTGGAATTAATTTGACTGTAAACACAAAGATATTAGTACAAAATACGTGACGTAGAAAGTAATAATTTCTTGGGTAGTTTGCAGTTTTAAAATTATGTTTTAAAATGGACTATCATATGCTTACCGTAACTTGAAAGTATTTCGATTTCTTGGCTTTATATATCTTGTGGAAAGGACGAAACACC), an initiating G nucleotide, the specific gRNA sequence, and the modified stem loop sequence of the sgRNA with a 3′ linker to the BamHI site (GTTTAAGAGCTAAGCTGGAAACAGCATAGCAAGTTTAAATAAGGCTAGTCCGTTATCAACTTGAAAAAGTGGCACCGAGTCGGTGCTTTTTTTCTCGAGTACTAGGATCCATTAGGCGGCCGCGTGGATAACCGTA). Specific gRNA sequences are listed in Supplementary Table 1.

### Lentiviral production & transduction

HEK293T cells were transfected with second generation lentiviral packaging plasmids using Lipofectamine 3000 transfection reagent in HEK293 growth medium (DMEM with 10% FBS). The medium was changed 16 h post-transfection, and viral supernatant was collected 2 and 3 days later. The supernatant was passed through a 0.45 μM filter and centrifuged at 20,000× *g* for 1.5 h at 4 °C. The concentrated virus was resuspended in DMEM and stored at -80 °C. All viruses were packaged independently, titrated and combined upon transduction. Lentiviral transduction of fibroblasts was performed in fibroblast medium supplemented with 4 μg/ml polybrene and 10 mM HEPES. Following the addition of lentivirus, cells were centrifuged for 90 min at 600× *g* and then incubated for an additional 4–6 h at 37 °C, after which the transduction medium containing lentivirus was replaced with fresh fibroblast medium.

### Fibroblast reprogramming & clonal iPSC generation

Before initiating reprogramming, human neonatal fibroblast cells (FTC0007) were transduced as described above with three lentiviruses: Tre3G, TetOn-dCas9-10XGCN4-P2A-mCherry and ScFV-GCN4-VP64-GFP. Transduced cells were treated with 1 μg/ml doxycycline for 24 h and were sorted in bulk using fluorescence-activated cell sorting (FACS) for expression of GFP (VP64) and mCherry (dCas9). Sorted fibroblast cells were again transduced with lentiviruses expressing indicated gRNAs. To initiate reprogramming, fibroblast medium supplemented with 1 μg/ml doxycycline was added to induce dCas9 expression. After 1 week, doxycycline treatment was discontinued, and the cells were passaged using Accutase and were seeded onto 0.1% gelatin-coated six-well plates in indicated reprogramming media with or without small molecules PD0325901 (0.4 μM, Abcam), CHIR99021 (1 μM, Abcam), thiazovivin (5 μM, Ryss Labs) and SB431542 (2 μM, Abcam). On week 2, cells were passaged and seeded onto Matrigel-coated six-well plates in iPSC media with or without small molecules PD0325901, CHIR99021 and Thiazovivin. iPSC medium is no longer supplemented with SB431542 at this stage. iPSC colonies were ready to sort by FACS on week 3.

### Flow cytometry & single cell sorting

To clone reprogrammed cells by FACS, the culture was dissociated and resuspended in FACS buffer (Hank's Balanced Salt Solution (HBSS) with 2% FBS, 10 mM HEPES and 1% penicillin/streptomycin). Antibodies were added to the cell suspension and were incubated for 30 min at 4 °C. The stained cells were rinsed and resuspended in FACS buffer for sorting using the BD FACS Aria II. The following antibodies were used at 1:20: TRA-1-81 APC-conjugated TRA-1-81 (BD Biosciences Pharmingen), BUV-395-conjugated SSEA-4 (BD Biosciences), and PE-conjugated CD30 (BD Biosciences). For flow cytometric analysis, cells were run on the BD LSRFortessa flow cytometer and analyzed using the FlowJo software.

### Alkaline phosphatase staining

Alkaline phosphatase staining was performed according to the manufacturer's protocol using the Leukocyte Alkaline Phosphatase Kit (Sigma-Aldrich). Briefly, reprogrammed cultures were fixed with 4% paraformaldehyde (PFA) for 30 s, rinsed and stained with the alkaline dye mixture, and then were incubated in the dark for 15 min. Stained cells were rinsed with DPBS and imaged.

### Immunofluorescence staining

Reprogrammed cultures were rinsed once with DPBS and fixed in 4% PFA in DPBS for 15 min at room temperature. Fixed cells were then permeabilized with 0.15% Triton-X-100 in DPBS for 30 min and blocked using 1% bovine serum albumin (BSA) for another 30 min at room temperature. Cells were stained with antibodies diluted in 1% BSA as follows: anti-OCT4 at 1:200 (SC-9081, Santa Cruz Biotechnology), anti-NANOG at 1:20 (AF1997, R&D Systems), anti-SSEA-4 at 1:100 (MAB4304; Millipore), anti-TRA-1-81 at 1:100 (MAB4381 Millipore), Alexa Fluor 488 donkey anti-mouse at 1:250 (A21202; Invitrogen), Alexa Fluor 555 donkey anti-rabbit, at 1:250 (A31572; Invitrogen), and Alexa Fluor 555 donkey anti-goat at 1:250 (A21432; Invitrogen). Immunofluorescence imaging was performed using a Zeiss Axio Observer inverted microscope and AxioVision SE64 imaging software v4.9.1 (Carl Zeiss).

### G-banded karyotype analysis

G-banded karyotype analysis of metaphase iPSCs was performed by the WiCell Research Institute.

### Directed *in vitro* tri-lineage differentiation

Directed differentiation of iPSCs into cells of the three germ layers was performed as monolayer cultures using the STEMdiff Tri-lineage Differentiation Kit according to the vendor's protocols (STEMCELL Technologies). Briefly, iPSCs were seeded in iPSC medium at densities specific for each lineage protocol in six-well plates. On day one, iPSC medium was switched to lineage-specific media and changed daily for 7 days (endodermal- and mesodermal-specific protocols) or 9 days (ectodermal protocol) before the cells were harvested for analysis. Differentiation was evaluated by RNA-Seq as described below.

### Teratoma assay

All study procedures were performed under local approved animal care and user guidelines. iPSCs were dissociated and resuspended in 100 μl cold Matrigel diluted at 1:1 in DMEM/F-12. Cell-Matrigel suspension was injected subcutaneously into each hind leg of NOD-SCID IL-2Rγ mice (the Jackson Laboratory) at a concentration of either 5 × 10^5^ or 2 × 10^6^ cells per injection. Mice were assessed weekly for physical evidence of teratoma formation at the site of injection. Mice were euthanized and teratomas were harvested for histological analysis when teratomas measured approximately 1 cm^3^ (8–10 weeks post injection). Harvested teratomas were fixed in 4% PFA and sectioned at four microns per section. Two sections were mounted per slide and stained with hematoxylin and eosin and were subsequently analyzed by a certified pathologist (Crown Bioscience).

### Gene expression analysis by RT-PCR

Total RNA was isolated using the RNeasy Plus Mini Kit (Qiagen). Quantitative PCR was performed using TaqMan RNA-to-CT 1-Step Kit (Applied Biosystems) and was analyzed on a StepOnePlus qPCR System and QuantStudio 3 qPCR System (Applied Biosystems). Results were normalized to GAPDH, HPRT1 and ribosomal protein S23 reference genes. Relative gene expression was quantified using a 2∧delta delta Ct method. Probe sets used are listed in Supplementary Table 2.

### Gene expression analysis by RNA-Seq

Total RNA was isolated as above and was quality measured by the Agilent High Sensitivity RNA ScreenTape (Agilent) on the Agilent 4200 TapeStation System (Agilent). RNA quantity was measured by using the NanoDrop 8000 Spectrophotometer (Thermo Fisher Scientific). 200 ng of total RNA was used per sample to generate the mRNA library using KAPA mRNA HyperPrep Kit (Roche) following the manufacturer's protocol. Poly(A) RNA was captured with magnetic oligo-dT beads to produce mRNA material fragmented to 200–300 bp in the presence of Mg2+ by incubating at 94 °C for 6 min. First strand synthesis was performed by synthesizing 1st strand cDNA with random primers incubated for 10 min at 25 °C, 15 min at 42 °C and 15 min at 70 °C. Second Strand Synthesis and A-tailing was performed by incubating samples for 30 min at 16 °C and 10 min at 62 °C. Adaptor Ligation was performed by ligating 3′-dTMP adapters to 3′-dAMP library fragments and incubating for 15 min at 20 °C. Two bead-based cleanups were performed at 0.63X and 0.7X volumes respectively. Final library was constructed by PCR amplification with the adapter ligated template incubated for 45 s at 98 °C; 15 cycles of 15 s at 98 °C, 30 s at 60 °C, 30 s at 72 °C; and 1 min at 72 °C. A 1X volume bead-based PCR cleanup was then performed to produce final library. Final libraries were quantified by using the Qubit dsDNA HS Assay kit (Invitrogen) on the Qubit™ Flex Fluorometer (Invitrogen). Library quality and size were measured using a high sensitivity D1000 ScreenTape (Agilent) on the Agilent 4200 TapeStation System (Agilent). Libraries were diluted to 4 nM and pooled evenly for high-throughput sequencing. Sequencing was performed on Illumina NextSeq 500 Instrument (Illumina) with 2 × 76 pair-end reads targeting a minimum of 10 million pair-end reads per sample. Raw sequencing data from Illumina's NextSeq 500/550 platform was converted to FASTQ format using bcl2fastq (Illumina). FASTQ files were prepared for alignment using TrimGalore. Salmon selective-alignment algorithm was used to generate per-gene count data from FASTQ files using the Hg38 reference genome. The DEseq2 package was used to perform differential gene expression analysis and variance stabilization transformation (VST). Variance-stabilized gene expression data were analyzed via hierarchical clustering and principal component analysis in R using the base package ‘stats’.

### Statistical data analysis

Statistical analysis was performed using StepOne Software v2.3 and QuantStudio Design and Analysis Software v1.4.1 (Applied Biosystems) and GraphPad Prism 8 statistical software. Statistical significance was defined at p < 0.05.

## Results

### CRISPRa successfully reprograms human fibroblasts into iPSCs in a process that can be enhanced by small molecules

**Figure 1.**
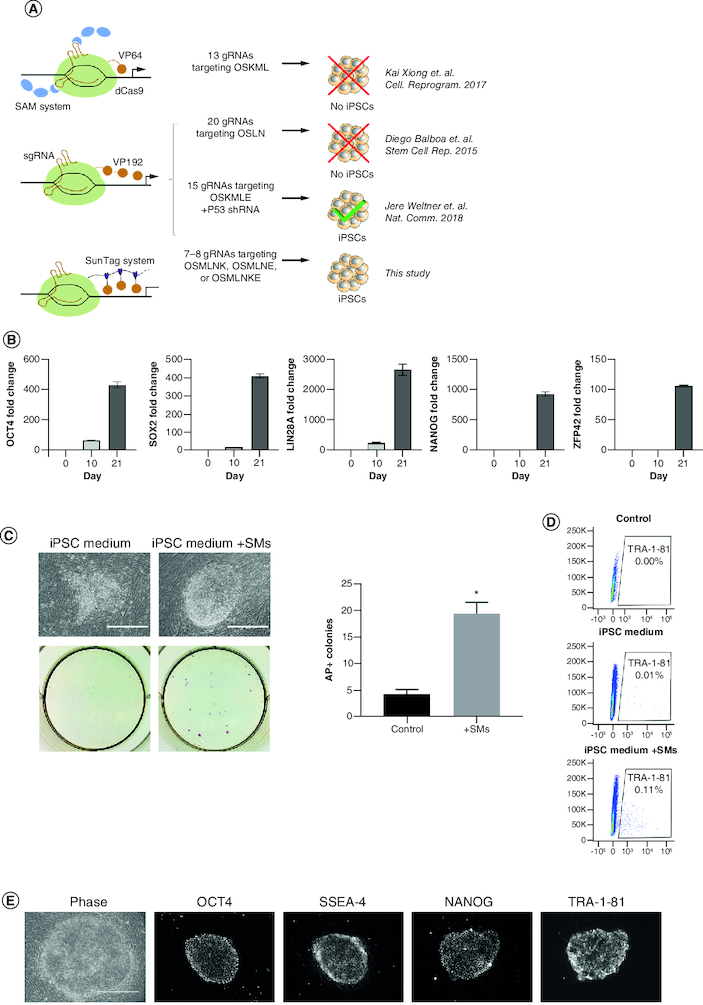
CRISPRa successfully reprograms human fibroblasts into iPSCs in a process that can be enhanced by small molecules. **(A)** Depictions of previous attempts aiming to reprogram human fibroblasts into iPSCs using CRISPRa. O, OCT4; S, SOX2; M, MYC; L, LIN28; N, NANOG; K, KLF4; E, EEA motif. **(B)** RT-qPCR expression analysis of indicated pluripotency genes on days 0, 10 and 21 post-induction. Bars represent the mean and standard error of the mean. **(C)** Representative phase images (top left) and alkaline phosphatase staining (bottom left) of iPSC colonies emerging 6 days post-induction. Scale bars are 400 μm. Number of AP positive colonies (right) emerging using reprogramming medium with and without small molecules (SM) at 6 days post-induction (n = 3). **(D)** Expression analysis of pluripotency surface marker TRA-1-81 by flow cytometry in reprogramming cultures in iPSC media with and without small molecules (SMs) 6 days post-induction. **(E)** Phase contrast and immunofluorescence staining of iPSC colonies for pluripotency markers OCT4, SSEA-4, NANOG and TRA-1-81 28 days post-induction. Scale bars are 400 μm. *p < 0.05.

Previous attempts to reprogram human fibroblasts into iPSCs by CRISPRa pointed toward the need to target larger sets of reprogramming genes, the use of more potent CRISPRa systems and/or multiple gRNAs per target, and the need to combine CRISPRa with miRNA or P53 knockdown (Figure 1A). We previously used the SunTag CRISPRa system to reprogram mouse fibroblasts to iPSCs with high efficiency [16]. To evaluate if the same SunTag system could be used to reprogram human fibroblasts, we targeted the same loci (Figure 1A & Supplementary Table 1) that led to the successful generation of human iPSCs by CRISPRa when combined with P53 knockdown [20]. However, to better elucidate the ability of CRISPRa alone in mediating reprogramming, we evaluated the generation of iPSCs by only using CRISPRa without P53 inhibitors or miRNAs and to avoid the derivation of genomically unstable iPSC lines. All loci were targeted using one gRNA per target except for *OCT4*, which was targeted with two gRNAs (a total of eight gRNAs to activate seven targets), as OCT4 has been shown to be critical during the cellular reprogramming process (Supplementary Table 1). We first verified the ability of each individual gRNA to activate the corresponding target gene. We observed marked transcriptional activation of *OCT4*, *SOX2*, *NANOG* and *LIN28* upon delivery of the corresponding individual gRNA (Supplementary Figure 1). The activation of *MYC* and *KLF4* was less evident as these genes are expressed in human fibroblasts and transcriptional activation may be masked by the non-reprogrammed cells. The combination of all the gRNAs led to a more robust activation of the targeted genes (Figure 1B), resulting in the formation of iPSC colonies which expressed the iPSC markers alkaline phosphatase and TRA-1-81 (Figure 1C & D). However, the number of iPSC colonies obtained was insufficient to support any screening application that requires clone selection and the derived colonies appeared to have morphology consisting of mixed composition of various cell types owing to the potential incomplete reprogramming process experience by cells in the colony. To enhance the efficiency of reprogramming and support complete reprogramming process, we evaluated the addition of small molecules known to enhance the efficiency of conventional reprogramming methods and to skew reprogramming toward naive/ground-state pluripotency [21,22,24] to the CRISPRa system (CRISPRa-SM). Reprogramming in the presence of the inhibitors of GSK3β (CHIR99021), MEK/ERK (PD0325901), TGF-β (SB431542) and Rho kinase (Thiazovivin) led to a more than tenfold increase in reprogramming efficiency (Figure 1C & D). The obtained CRISPRa-SM colonies exhibited typical and uniform iPSC morphology and expressed iPSCs markers (Figure 1D & E).

### CRISPRa-SM iPSC clonal lines exhibit naive pluripotency & maintain genomic stability

**Figure 2.**
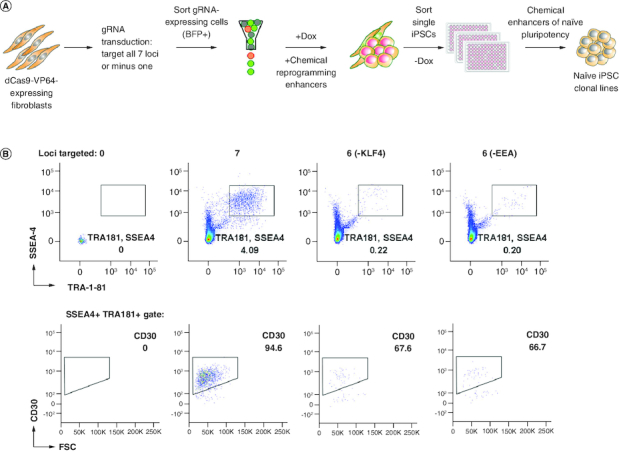
Generation of feeder-free single cell-derived naive-like CRISPRa iPSC lines. **(A)** Schematic representation of reprogramming platform combining the SunTag CRISPRa system, optimized stage-specific naive iPSC media, and single-cell sorting by FACS. **(B)** Flow cytometry analyses of three reprogrammed cultures selected for single-cell cloning by sorting. Other conditions were not sorted as iPSC markers were not detected.

To determine the genes required for a successful CRISPRa-SM mediated reprogramming and reduce the requirement and complexity of multiple-loci targeting, one gRNA was dropped at a time (Figure 2A). No iPSC colonies were observed when individual gRNAs for *OCT4*, *SOX2*, *LIN28*, *NANOG* and *MYC* were excluded, indicating that the activation of these genes is required. The requirement for the MYC gRNA is intriguing given that we did not observe significant activation of MYC above overall RNA expression levels (Supplementary Figure 1). It is possible that we missed the timing for detection of MYC activation by CRISPRa or activation level was hard to discern since human fibroblasts also express *MYC* at basic levels. IPSC colonies were observed when the individual *KLF4* and EEA-motif gRNAs were excluded albeit at markedly lower levels (96 and 97% reduction in SSEA4, TRA-1-81, CD30-expressing iPSCs, respectively, Figure 2B). To generate clonal CRISPRa iPSC lines, we utilized our naive iPSC generation platform for enhanced reprogramming, and iPSC cloning and expansion (Figure 2A) [^21-23^]. First, fibroblast cells enriched for Dox-inducible dCas9 were transduced with lentiviruses expressing gRNAs (all or without *KLF4*/EEA gRNA) and blue fluorescent protein (BFP). Seven days post induction, the reprogrammed cultures were expanded without Dox. To isolate and clone the successfully reprogrammed cells, the iPSCs were sorted at a clonal density into 96-well plates using the previously defined iPSC markers: SSEA4, TRA-1-81 and CD30 (Figure 2 & Supplementary Figure 2) [23]. Individual iPSC colonies derived from single cells were expanded in our small molecule-supplemented naive iPSC medium under feeder-free conditions and cryopreserved for further characterization.

**Figure 3.**
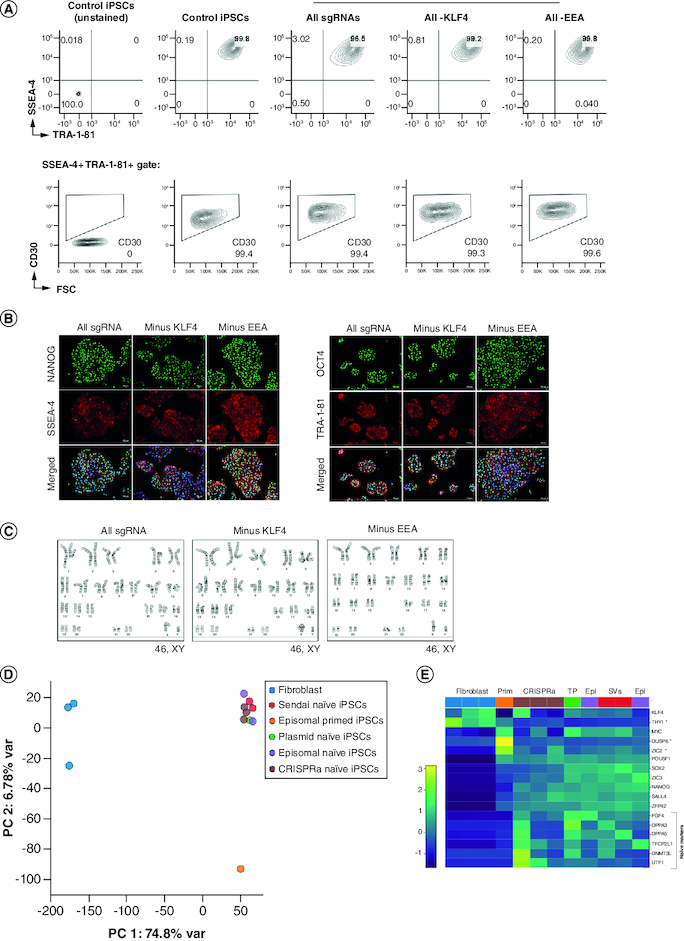
CRISPRa-SM iPSC clonal lines exhibit naive pluripotency and maintain genomic stability. **(A)** Flow cytometric analysis of CRISPRa iPSC clones for pluripotency surface markers SSEA-4, TRA-1-81 and CD30. Control iPSCs were generated using plasmid-based reprogramming methods. **(B)** Immunofluorescence staining for pluripotency markers NANOG, SSEA-4, OCT4, and TRA-1-81. Scale bars are 100 μm. **(C)** G-banded karyotype analysis of CRISPRa-derived iPSC clones. **(D & E)** Gene expression analysis by RNA-seq. Principal component analysis of parental fibroblasts and derived iPSC lines generated using various reprogramming methods (CRISPRa, episomal vectors (Epi), Sendai viruses (SVs) and transient plasmid-based (TP). **(D)** Heat map depicting expression of naive (bracket), primed (starred) and shared pluripotency genes in parental fibroblasts and derived iPSC lines generated using indicated reprogramming methods. **(E)** All iPSC lines were generated in naive iPSC medium except for the indicated control iPSC line which was maintained in primed iPSC medium (E8 medium, Prim).

Cryopreserved CRISPRa-SM iPSCs lines recovered well post-thaw and exhibited a homogenous and stable expression of iPSC surface markers as well as the pluripotency master regulators NANOG and OCT4 (Figure 3A & B). As expected, dCas9 was not expressed in the iPSC lines (expanded in absence of Dox) indicating that pluripotency is no longer dependent on CRISPRa (Supplementary Figure 3). The CRISPRa-SM iPSC lines maintained a normal karyotype after extended culture, demonstrating genomic stability (Figure 3C). To compare iPSCs generated using CRISPRa and other conventional reprogramming methods, we evaluated the global gene expression by RNA-Seq. CRISPRa-generated naive iPSC lines clustered together in the principal component analysis with other naive iPSC lines generated using episomal plasmids, Sendai viruses, or transient plasmids (Figure 3D). All iPSC lines clustered separately from the parental fibroblast cells, and naive iPSC lines clustered separately from the iPSC control line cultured in primed iPSC medium. Like naive iPSC lines generated using other methods, the CRISPRa-SM naive iPSC lines expressed naive and canonical pluripotency genes (Figure 3E). Overall, the CRISPRa-SM iPSC lines including those generated without activating the endogenous *KLF4* or genes with regulatory sequences enriched with the EEA-motif maintained typical iPSC morphology, global gene expression, naive pluripotency state and genomic stability.

### CRISPRa-SM iPSC lines have a similar pluripotent potential to that of conventional iPSCs

**Figure 4.**
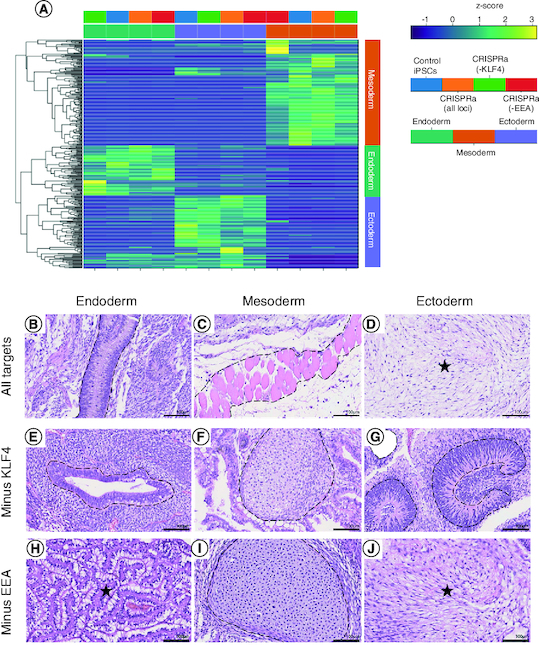
CRISPRa-SM iPSC lines have a similar pluripotent potential to that of conventional iPSCs. **(A)** *In vitro* tri-lineage differentiation of CRISPRa-generated and plasmid-generated (control) iPSC lines analyzed by RNA-seq show successful differentiation into the three germ layers. **(B–J)** Histological analysis of tissue sections of CRISPRa iPSC-derived teratomas harvested at 6–8 weeks; hematoxylin and eosin staining. Representative endodermal, mesodermal and ectodermal tissues present in excised and sectioned teratoma. **(B)** Columnar epithelium. **(C)** skeletal muscle. **(D)** Neuroepithelium. **(E)** Ductal structure; columnar epithelium. **(F)** Cartilage. **(G)** Neuroepithelium. **(H)** Gastroinstestinal mucus-secreting columnar epithelium. **(I)** Cartilage. **(J)** Neural tissue. Scale bars are 100 μm.

To evaluate their pluripotent potential, the tri-lineage differentiation capacity of CRISPRa-SM iPSC lines was tested using three directed differentiation protocols. Following differentiation of CRISRPa iPSC lines to endoderm, mesoderm and ectoderm lineages, differentiated cultures were evaluated by RNA-seq analysis. All the CRISPRa iPSC lines including those generated without using the *KLF4* or EEA-motif gRNAs exhibited robust tri-lineage differentiation as evident by the expression of lineage markers specific to the corresponding differentiation protocol (Figure 4A). No marked differences were observed in the tri-lineage differentiation potential of the CRISPRa-SM iPSC lines compared with iPSCs generated by the conventional plasmid-based reprogramming method. The pluripotency of the CRISPRa iPSC lines was further evaluated using the *in vivo* teratoma assay, the most stringent pluripotency test applicable to human PSCs. CRISPRa-SM iPSC lines were subcutaneously injected into immunocompromised NSG mice. The developed teratomas were collected 8–10 weeks post-injection for histological analysis. All the CRISPRa-SM iPSC lines tested generated with or without the use of the *KLF4* or the EEA-motif gRNAs showed robust tri-lineage differentiation as evident from the observed mature endodermal, mesodermal and ectodermal structures (Figure 4B–J). These results confirm that the SunTag-based CRISPRa-SM is capable of reprogramming human fibroblasts into iPSCs with robust pluripotent potential that is equivalent to that of iPSCs generated by conventional methods which most commonly include forced transgene expression.

## Discussion

We previously generated iPSCs from murine fibroblasts using the SunTag CRISPRa system [16]. In this study, we used the SunTag system to activate the endogenous expression of reprogramming genes and generated iPSCs from human fibroblasts. However, the generation of human iPSCs required the simultaneous activation of multiple reprogramming genes, displayed a low efficiency of cellular reprogramming with the few colonies derived consisting of a heterogenous population of various cellular morphologies, indicating higher epigenetic barriers that must be overcome for successful human iPSC generation. Reprogramming of human fibroblasts into iPSCs required the activation of at least *OCT4*, *SOX2*, *MYC*, *LIN28*, *NANOG* and *KLF4*. Activating *KLF4* can be replaced with the activation of the EEA-motif enriched in the promoters of genes expressed during embryo genome activation, consistent with previous reports [17,20]. There were no marked differences between the CRISPRa iPSC lines generated with or without using the *KLF4* or EEA-motif gRNAs. The iPSC generation by the CRISPRa system was markedly improved by the addition of small molecule inhibitors of relevant developmental signaling pathways [16]. Stage-specific application of small molecules allowed for the seamless generation of iPSC lines from single cells with efficiency that is similar to that observed with other reprogramming methods [21,23]. The application of CRISPRa-SM, consisting of small molecule inhibitors of GSK3β, MEK/ERK, and Rho kinase in combination with an optimized milieu of cytokines bias the generation of iPSCs toward naive/ground-state pluripotency [22]. Accordingly, the CRISPRa-SM iPSCs expressed naive iPSC markers and showed related high-quality attributes such as high clonogenicity, stability after single cell and feeder-free passage, and robust differentiation potential.

CRISPRa was required only transiently for the generation of iPSCs, and the reprogrammed culture was stable following the removal of Dox. The generated iPSC lines maintained a stable pluripotency without Dox and showed independence from continuous modulation by CRISPRa. The iPSC lines maintained homogeneous and stable expression of iPSC markers as well as genomic stability after extended culture. The CRISPRa-SM iPSC lines were indistinguishable in morphology, phenotype or global gene expression from iPSC lines generated using conventional reprogramming methods. All CRISPRa-SM iPSC lines showed robust tri-lineage specification and differentiation *in vitro* and using the teratoma murine model indicating that CRISPRa led to bona fide reprogramming of human fibroblasts into iPSCs.

Human iPSCs have been generated previously using CRISPRa activation, but this required the use of P53 knockdown by shRNA and/or miR-302/367 miRNA cluster [17,20]. Activation of endogenous reprogramming also required the use of multiple gRNAs per targeted locus, perhaps due to the use of a simpler CRISPRa system (dCas9-VP192). Surprisingly, the dCas9-VP192 was more effective in activating *OCT4* than the more complex trimethoprim stabilized dCas9-VP192 fused with P65-HSF1 activator domain under a Dox-inducible promoter [20]. In this study, we show that using the SunTag system alone with a minimal number of gRNAs was sufficient in reprogramming human fibroblasts into iPSCs.

## Conclusion

Overall, we show that the CRISPRa-SM system alone led to the successful reprogramming of human fibroblasts into iPSCs with reduced dependency on multiplexed activation of multiple human loci, eliminated the need for P53 modulation that promotes genomic instability and the reduction for the need of oncogene activation, namely KLF4, while producing high quality iPSCs for various therapeutic applications.

## Supplementary Material

Supplementary Figures S1-S3 and Tables S1-S2
