# Supplementary material for: A chemical approach facilitates CRISPRa-only human iPSC generation and minimizes the number of targeted loci required
Source: Future Sci OA. 2024 May 15;10(1):FSO964. doi: 10.2144/fsoa-2023-0257 (PMC11137772; doi:10.2144/fsoa-2023-0257)
Supplement: Supplementary Figures S1-S3 and Tables S1-S2 [file IFSO_A_2340855_SM0001.zip › Table_S1.docx]

**Table S1. Guide RNA sequences.**

| Target | Guide RNA sequence |
| --- | --- |
| *OCT4* promoter | GGGGGAGAAACTGAGGCGA |
| *OCT4* promoter | TCTGTGGGGGACCTGCACTG |
| *SOX2* promoter | GTGGCTGGCAGGCTGGCTCT |
| *KLF4* promoter | GCTGCCATAGCAACGATGGA |
| *MYC* promoter | GGTTCCCAAAGCAGAGGGCG |
| *NANOG* promoter | GATTAACTGAGAATTCACAA |
| *LIN28A* promoter | GTGTCAGAGACCGGAGTTGT |
| EEA motif | CCCAGCACTTTGGG |
