# Supplementary material for: A chemical approach facilitates CRISPRa-only human iPSC generation and minimizes the number of targeted loci required
Source: Future Sci OA. 2024 May 15;10(1):FSO964. doi: 10.2144/fsoa-2023-0257 (PMC11137772; doi:10.2144/fsoa-2023-0257)
Supplement: Supplementary Figures S1-S3 and Tables S1-S2 [file IFSO_A_2340855_SM0001.zip › Table_S2.docx]

**Table S2. List of TaqMan assays used.**

| TaqMan assay | Part number |
| --- | --- |
| *OCT4 (POU5F1)* | Hs04260367_gH |
| *SOX2* | Hs00602736_s1 |
| *KLF4* | Hs00358836_m1 |
| *MYC* | Hs99999003_m1 |
| *NANOG* | Hs02387400_g1 |
| *LIN28* | Hs00702808_s1 |
| *ZFP42* | Hs00399279_m1 |
| *GAPDH* | Hs02786624_g1 |
| *RP23* | Hs01374150_g1 |
| *HPRT1-PL* | Hs02800695_m1 |
