# Supplementary figures and images for: A chemical approach facilitates CRISPRa-only human iPSC generation and minimizes the number of targeted loci required
Source: Future Sci OA. 2024 May 15;10(1):FSO964. doi: 10.2144/fsoa-2023-0257 (PMC11137772; doi:10.2144/fsoa-2023-0257)

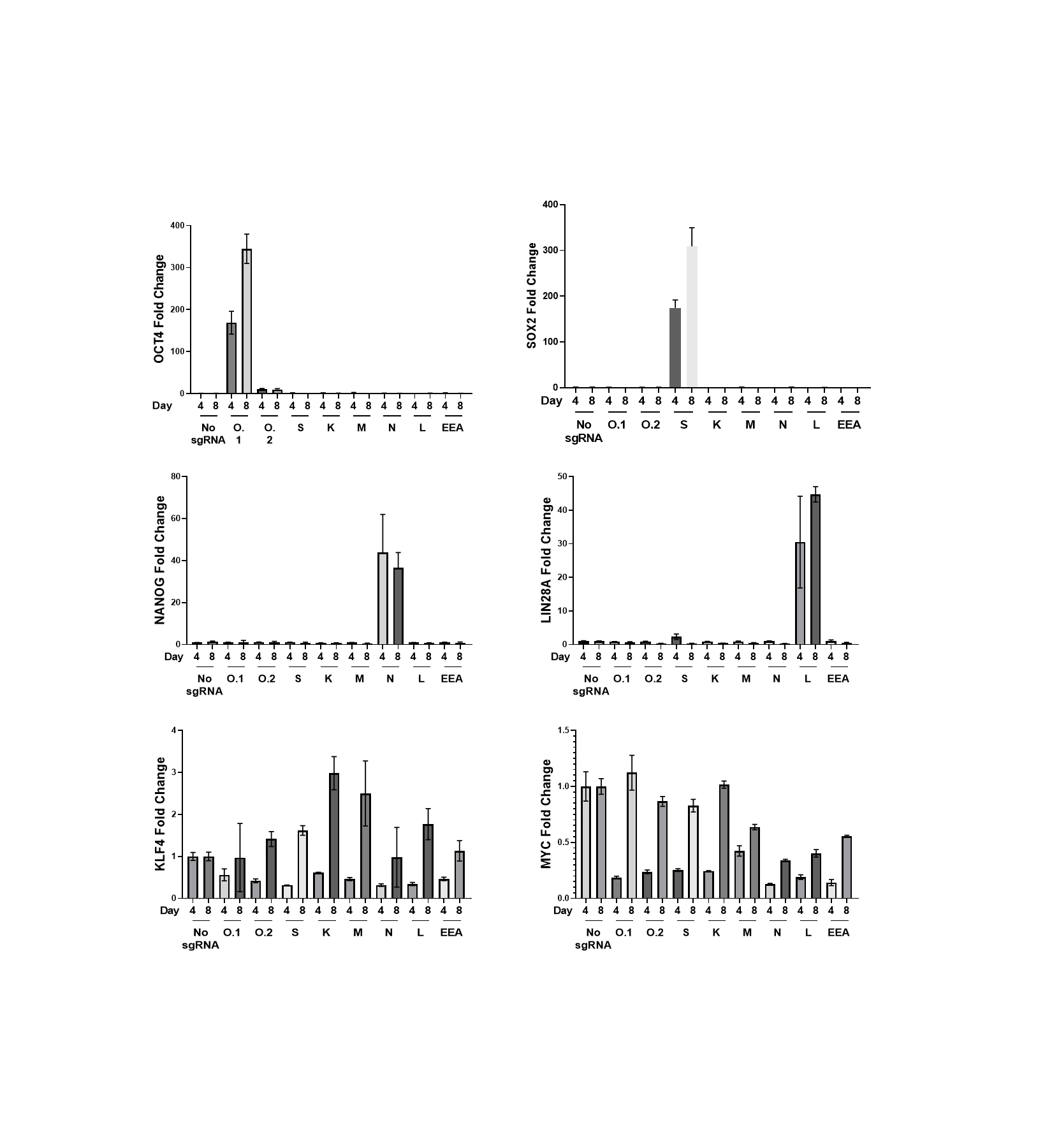

Supplement: Supplementary Figures S1-S3 and Tables S1-S2 [file IFSO_A_2340855_SM0001.zip › Figure_S1.tif]

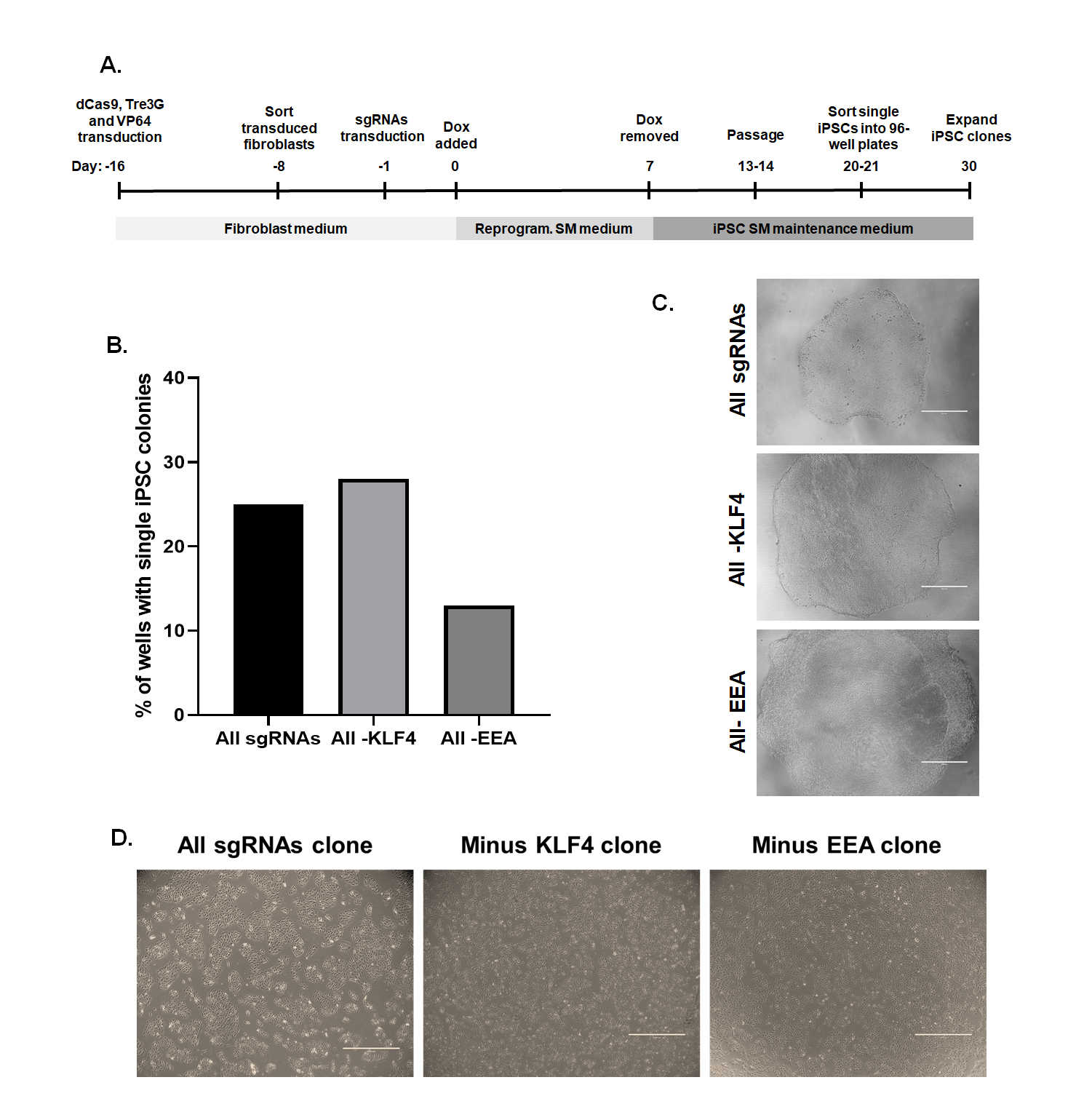

Supplement: Supplementary Figures S1-S3 and Tables S1-S2 [file IFSO_A_2340855_SM0001.zip › Figure_S2.jpg]

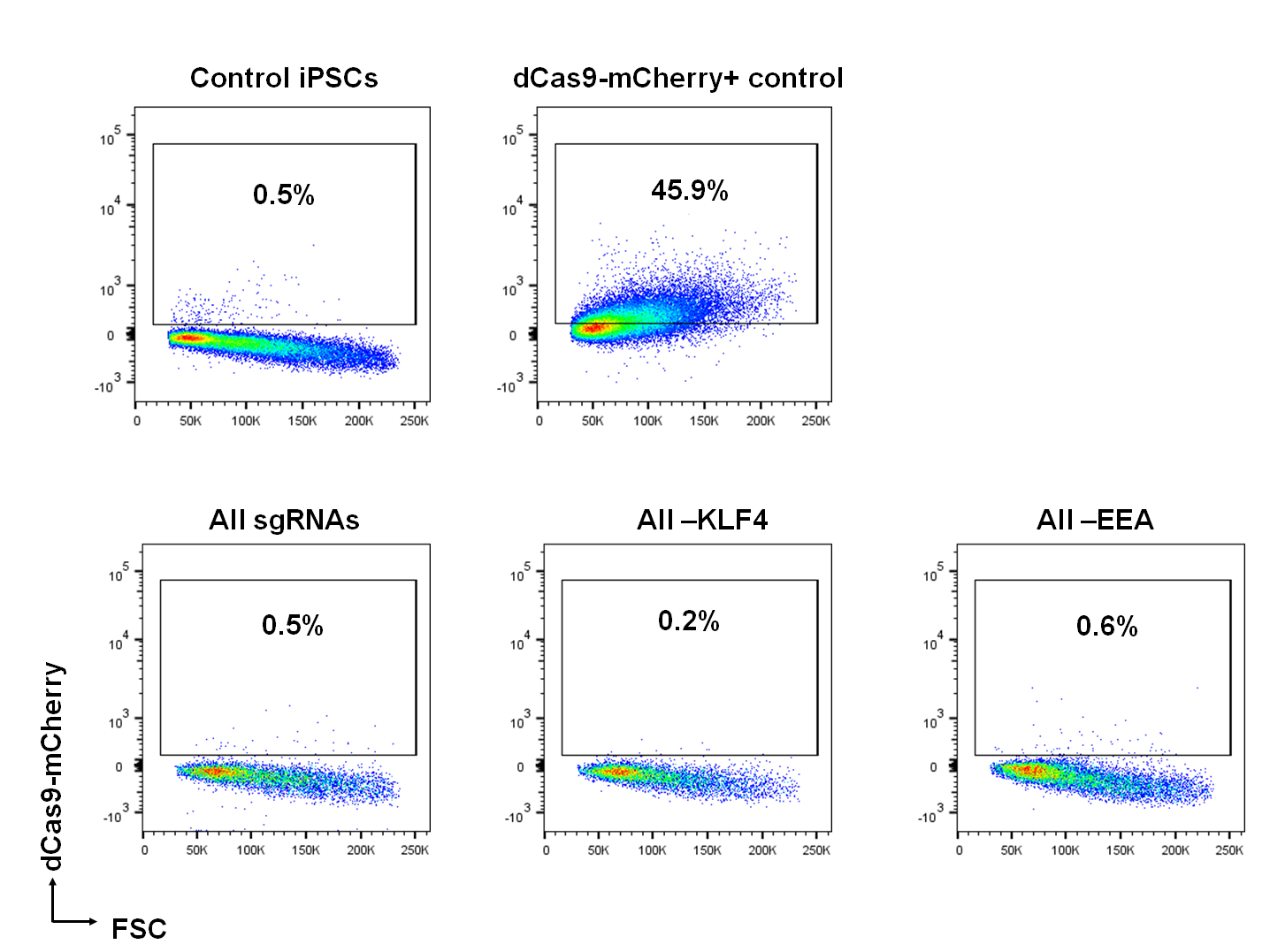

Supplement: Supplementary Figures S1-S3 and Tables S1-S2 [file IFSO_A_2340855_SM0001.zip › Figure_S3.tif]
